# Design of the DYNAMO study: a multi-center randomized controlled trial to investigate the effect of pre-thickened oral nutritional supplements in nursing home residents with dysphagia and malnutrition (risk)

**DOI:** 10.1186/s12877-020-01947-4

**Published:** 2020-12-14

**Authors:** Viviënne A. L. Huppertz, Nick van Wijk, Laura W. J. Baijens, Lisette C. P. G. M. de Groot, Ruud J. G. Halfens, Jos M. G. A. Schols, Ardy van Helvoort

**Affiliations:** 1 Department Respiratory Medicine, Maastricht University, Nutrition and Translational Research in Metabolism (School NUTRIM), Maastricht, the Netherlands; 2 Danone Nutricia Research, Utrecht, the Netherlands; 3 Department of Otorhinolaryngology, Head and Neck Surgery, Maastricht University Medical Centre, Maastricht, the Netherlands; 4 Maastricht University Medical Centre, School for Oncology and Developmental Biology – GROW, Maastricht, the Netherlands; 5 Division of Human Nutrition and Health, Wageningen University and Research Centre, Wageningen, the Netherlands; 6 Department Health Services Research, Maastricht University, Care and Public Health Research Institute (School CAPHRI), Maastricht, the Netherlands

**Keywords:** Geriatrics, Malnutrition, Dysphagia, Pre-thickened oral nutritional supplement, Nutritional status

## Abstract

**Background:**

Oropharyngeal Dysphagia (OD) and malnutrition are frequently reported conditions in nursing home residents, and are often interrelated. Best care for dysphagic residents with, or at risk of, malnutrition should target adequate nutritional intake and the safety and efficacy of swallowing. The effect of oral nutritional supplements (ONS) suitable for nursing home residents with concurrent OD and malnutrition (risk) on nutritional status has not been investigated before. The current study aims to investigate the effect of daily use of a range of pre-thickened ONS on the body weight of nursing home residents with OD and malnutrition (risk) compared to standard OD and nutritional care.

**Methods / design:**

The DYNAMO study is a randomized, controlled, multi-center, open label trial with two parallel groups. Study participants will be recruited in nursing homes of several care organizations in the south of the Netherlands. Study duration is 12 weeks. Residents in the control group will receive standard OD and nutritional care, and residents in the test group will receive standard OD and nutritional care with extra daily supplementation of pre-thickened ONS.

The main outcome parameter is the difference in body weight change between the control and test groups. An a priori estimation of the required sample size per group (control / test) totals 78. Other outcome parameters are differences in: nutritional intake, health-related quality of life, OD-specific quality of life, activities of daily living, vital signs, and blood nutrient and metabolite levels.

**Discussion:**

Regular ONS could address the nutritional needs of nursing home residents with malnutrition (risk), but might be too thin and unsafe for residents with OD. Pre-thickened ONS is suitable for residents with OD. It offers the advantage of being a ready-to-use amylase-resistant product available in several consistencies which are able to increase swallowing efficacy and safety. The DYNAMO study is the first to investigate the effects of pre-thickened ONS on nutritional status in nursing home residents with concurrent OD and malnutrition (risk).

**Trial registration:**

Netherlands Trial Register (NTR): NTR NL7898. Registered 24 July 2019, https://www.trialregister.nl/trial/7898

## Background

Malnutrition is frequently reported in nursing home populations, although prevalence rates vary considerably between studies, depending on the diagnostic criteria used [1, 2]. A recent study in Dutch nursing homes revealed that 20% of the residents was at risk of malnutrition [3]. A good nutritional status is essential for nursing home residents to maintain overall health, recover from disease, and optimize health-related quality of life (HRQoL) [4, 5]. Conversely, poor nutritional status, e.g. unintentional weight loss, is associated with higher morbidity [6] and mortality rates [7, 8], and lower HRQoL [9].

Nursing home residents are susceptible to malnutrition due to aging-related diseases, physiological changes in body composition and metabolism [10], care dependency [11], and swallowing impairment or eating difficulties [3]. Oropharyngeal dysphagia (OD) affects the efficacy and safety of swallowing as a result of aging-related functional bodily impairment or pathophysiological changes due to dementia, Parkinson’s disease, or stroke. A Dutch prevalence study using a standardized questionnaire revealed a prevalence rate of 12% for subjective OD symptoms [12]. Dysphagic residents are at high risk of aspiration (pneumonia), dehydration, malnutrition, and weight loss [13]. A previous study by Carrión et al. [14] described a bidirectional relationship between OD and malnutrition, with 51% of the residents having OD and malnutrition (risk). While OD affects a residents’ ability to eat, the consequences of malnutrition affect the ability to swallow; it is a vicious circle.

Nutritional care for dysphagic nursing home residents with malnutrition (risk) should thus simultaneously target adequate nutritional intake and the safety and efficacy of swallowing. Current European and Dutch guidelines for the management of OD and malnutrition suggest food fortification, oral nutritional supplementation (ONS), texture modification of solid food products, and/or thickening of liquids [15–17]. Thick, more viscous fluids tend to flow more slowly, and the assumption is that slower flow improves upper aerodigestive bolus control during swallowing. Studies suggest that increasing fluid thickness, i.e. the viscosity of the fluid, results in increased safety of swallowing, i.e. less aspiration risk [18, 19]. A more severe swallowing impairment often requires higher levels of fluid thickness to prevent aspiration [8].

The thickness of regular ONS is relatively low and is therefore not suitable for the majority of residents with OD and malnutrition (risk). The thickness of regular ONS can be further increased with a thickening powder, which is time consuming and offers an insufficient guarantee of obtaining the desired ONS thickness. Compared to regular ONS, pre-thickened ONS offers the advantage of being a ready-to-use, amylase-resistant product available in several consistencies (from drinkable to spoonable), which are able to increase swallowing efficacy and safety. A recent study showed increased product compliance and user-convenience with pre-thickened ONS compared to manually thickened ONS, with similar gastro-intestinal tolerability [20].

Benefits of ONS on nutritional status in nursing home residents with malnutrition (risk) were described previously [21–24], however no studies exist on the effect of pre-thickened ONS on swallowing in dysphagic nursing home residents. Scientific evidence on the effect of pre-thickened ONS for nutritional care in dysphagic nursing home residents having malnutrition (risk) is needed. The DYNAMO^1^ study aims to investigate the effect of daily use of a range of pre-thickened ONS for 12 weeks on the body weight of nursing home residents with OD and malnutrition (risk) compared to standard OD and nutritional care.

## Methods and design

### Study design

**Fig. 1:**
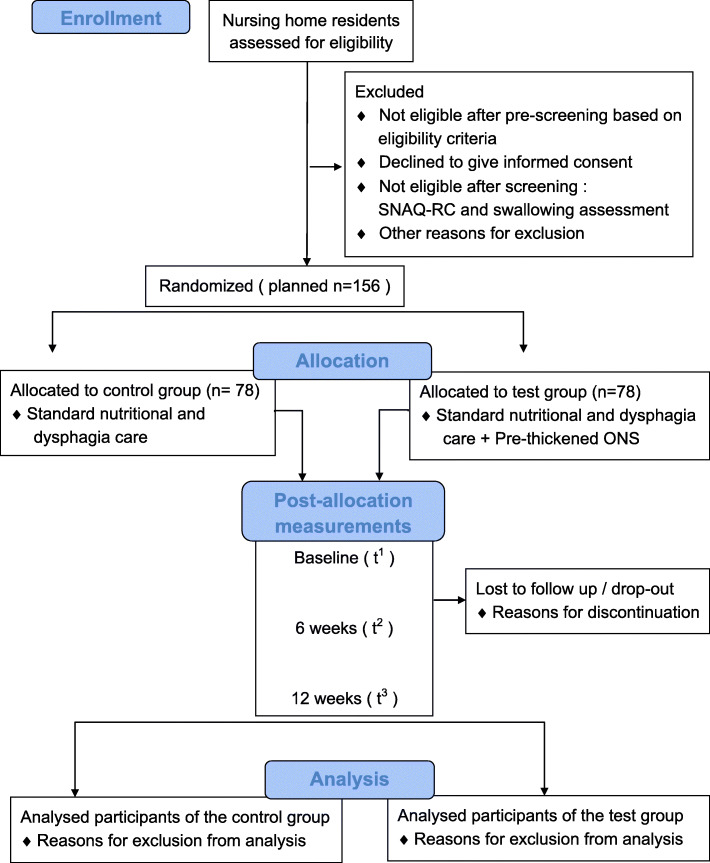
Flow diagram of participants of the DYNAMO study. Abbreviations: short nutritional assessment questionnaire for residential care (SNAQ-RC), oral nutritional supplementation (ONS)

The current study is a randomized, controlled, multi-center, open label (no blinding) trial with two parallel groups. The study duration is 12 weeks, preceded by an enrolment period including pre-screening, informed consent, and definitive screening. A flow diagram of participants is shown in Fig. 1. If eligible, nursing home residents are randomly allocated into the control or test group directly upon screening (1:1 ratio). Nursing home residents in the control group will receive standard OD and nutritional care, and will be compared to residents in the test group receiving standard OD and nutritional care with extra daily supplementation of the test product. Measurements are performed at baseline (t_1_), 6 weeks (t_2_), and 12 weeks (t_3_). The schedule of enrolment, intervention and assessments can be found in Table 1.

**Table 1:**
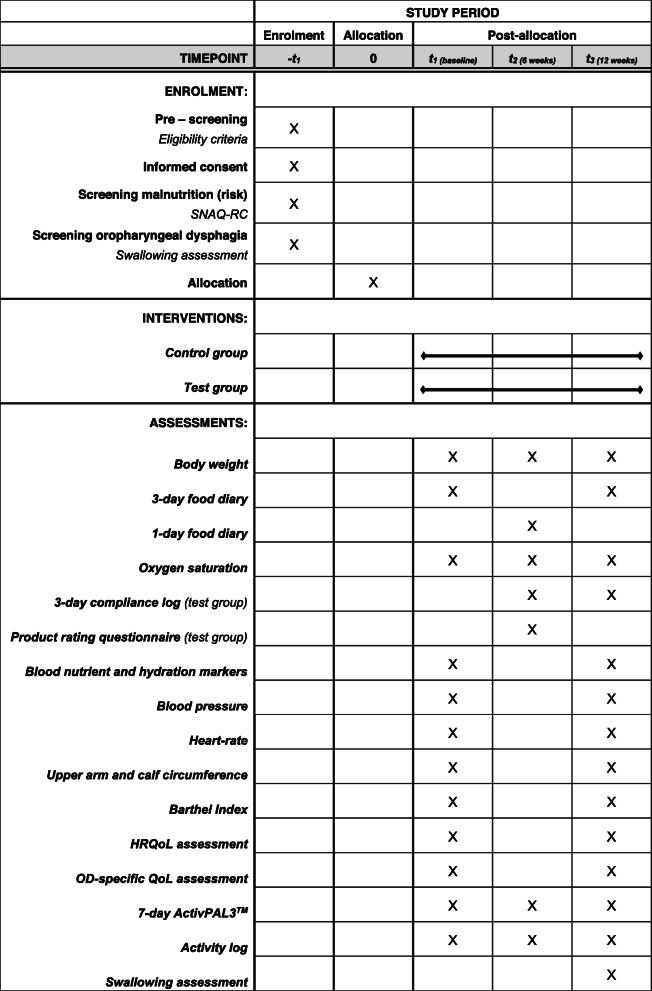
Schedule of enrolment, intervention and assessments. Abbreviations: short nutritional assessment questionnaire for residential care (SNAQ-RC), health-related quality of life (HRQoL), oropharyngeal dysphagia-specific quality of life (OD-specific QoL)

### Study population

The study will be carried out in nursing homes of several care organizations in the south of the Netherlands. Participants of the study will be nursing home residents that meet the eligibility criteria (Table 2). All eligibility criteria, except for OD and malnutrition (risk), will be verified by the nursing home physician using the electronic patient file.

**Table 2:** Eligibility criteria for the DYNAMO study

Inclusion criteria	Exclusion criteria
• Permanent admission to or living in a somatic or psychogeriatric ward • ≥ 65 years of age • Risk of malnutrition, or malnutrition • Oropharyngeal dysphagia • Informed consent	• Daily use of protein- and energy-containing ONS over a period of four weeks prior to screening • Daily use of enteral or parenteral nutrition over a period of four weeks prior to screening • Renal disease requiring dialysis • Both lower legs amputated • Known allergy or intolerance to any ingredient of the test product • Participation in any other study within 6 weeks prior to or after randomization • Known cachexia • Uncertainty about the willingness or ability of the resident to comply with the protocol requirements

### Pre-screening and screening

Nursing home residents who meet any of the exclusion criteria (Table 2) will be excluded during pre-screening. Pre-screening will be conducted by a member of the trained study team.

The validated short nutritional assessment questionnaire for residential care (SNAQ-RC) [25] will be used as the screening tool for malnutrition (risk). The SNAQ-RC includes body mass index (BMI), unintended body weight loss, care dependency in feeding, and loss of appetite.

A swallowing assessment will be carried out by a speech and language therapist (SLT) to screen nursing home residents for clinically relevant signs of OD. The swallowing assessment consists of four parts that are illustrated and clarified in Table 3. Based on this swallowing assessment, the SLT determines the presence of OD and appropriate thickness of the test product.

**Table 3:** Content of the swallowing assessment. Abbreviations: 3 oz water swallow test (3-oz WST), Water Swallow Test (WST), McGill Ingestive Skills Assessment (MISA)

	Assessment	Assessment details
**Part I**	3-oz WST [26]	• Resident is asked to drink a glass of 3 oz. of water at room temperature while seated • SLT assesses whether glass is emptied without interruption and without signs of impaired swallowing such as impaired oropharyngeal bolus transit, oral cavity pooling, coughing, drooling, and voice change
**Part II**	Structured observation of oral intake	• Observation during a meal including solid as well as liquid products • SLT focuses on maintenance of body posture, self-feed ability, and signs of impaired swallowing, and observations will be documented
**Part III**	WST with different thickness levels	• Water boluses with different thickness levels are prepared: water + thickening powder • Resident is asked to swallow at least three subsequent sips of the thickened bolus. • SLT determines the appropriate thickening of liquids for safe swallowing, i.e. the thickness at which the resident is able to swallow without showing signs of impaired swallowing
**Part IV**	Modified version of the MISA [27]	• Evaluation of functional ingestive skills of older persons: maintenance of symmetrical body posture and adequate head position for feeding, ability to grasp and use utensil and cup/glass functionally, appropriately-sized mouthfuls, voice quality after drinking, airway clearance after drinking, quantity of food remaining in mouth after swallowing, location of food remaining in mouth after swallowing, voice quality after earing, airway clearance after solids, ability to eat regular solids, soft solids, puree and pudding, and ability to drink water, thin juices, nectar, honey and pudding

### Randomization

Eligible nursing home residents will randomly be allocated to the control or test group using the online randomization tool ALEA™ (ALEA Clinical by FormsVision, Abcoude, the Netherlands). Block randomization will be performed with 1:1 allocation based on nursing home (site) and type of resident (legally capacitated vs. legally incapacitated to act for oneself) and varying block sizes of two and four.

### Interventions

#### Standard OD and nutritional care

Both the control and test group will receive standard OD and nutritional care. Standard nutritional care in the current study is in line with the Dutch guidelines for malnutrition [15] and according to the most recent guideline on nutrition in geriatrics of the European Society for Clinical Nutrition and Metabolism (ESPEN) [17]. The control and test group receive dietary counseling and food fortification with enrichment of meals and provision of snacks in between meals. Regular ONS, possibly manually thickened to a safe thickness level, is only provided to the control group residents if food fortification is not sufficient to increase dietary intake and reach nutritional goals. Standard OD care is defined as the routine practice in each nursing home, with references to the Dutch guidelines for OD [16]. 

#### Test product

The test group will receive pre-thickened ONS (the test product) in addition to the standard OD and nutritional care without pre-thickened ONS. The commercially available test product (Nutilis® Complete, Nutricia, Zoetermeer, the Netherlands) is a food for special medical purposes and is provided in 125 mL bottles or 125 g pots. It is intended for dietary management of disease-related malnutrition, and is a high energy, nutritionally complete pre-thickened supplement suitable for patients with swallowing difficulties. Previously conducted clinical and non-clinical studies did not reveal any safety concerns [28–30].

This ONS is pre-thickened to 4 standardized consistencies (i.e. with increasing thickness) that suits the different severity levels of OD, in accordance with the British Dietetic Association (BDA) guidelines [31]. The target viscosity (i.e. thickness defining its resistance to flow) of the pre-thickened ONS is 65, 450, 1200, and 3000 mPa*s at a shear rate of 50 s^− 1^ and a temperature of 20 °C. Table 4 shows an overview of the available flavors, energy density (kCal/100 mL or g), viscosity (mPa.s), BDA stage [31], and corresponding IDDSI level (International Dysphagia Diet Standardization Initiative) for each stage of the pre-thickened ONS [32].

**Table 4:** Overview of the test product range

Pre-thickened ONS range	Flavors	Energy density ***(kCal/100 mL or 100 g)***	Viscosity ***(mPa***^a^***s)***	BDA^a^ stage	Corresponding IDDSI^b^ Level
**Drink**	vanilla / strawberry	200	65	n/a	2
**Drink**	vanilla / strawberry / lemon tea / mango passion fruit	245	450	1	3
**Crème**	vanilla / strawberry / chocolate	245	1200	2	3
**Fruit**	strawberry / apple	138	3000	3	4

^a^ Severity levels of OD in accordance with the British Dietetic Association (BDA) guidelines [31]

^b^ Framework of the International Dysphagia Diet Standardization Initiative (IDDSI) [32]

For the test group, a trained SLT and a dietician will provide a patient-tailored recommendation on the safest thickness level of liquids for swallowing and for the amount of nutritional supplementation (≥ 500 kCal or 2–3 units of test product per day).

#### Blinding

Investigating the effect of the test product on top of standard OD and nutritional care precludes a (iso-caloric) control. Therefore, blinding is not possible, and all assessors need to be informed of the intervention plan of the resident. The study coordinator will be involved in the data collection process as well as in data analysis, which also precludes blinded data analysis.

### Study outcomes

The primary outcome of the DYNAMO study is the difference in body weight change between the control and test group from baseline to final measurements after 12 weeks of intervention.

Secondary outcomes are changes in nutritional intake, HRQoL, OD-specific QoL, and patient compliance to the test product. In addition, a food diary, oxygen saturation, blood nutrient and hydration markers, blood pressure, heart-rate, upper arm and calf circumference, Barthel Index, 7-day activPAL3™, activity log, rating of the test product and dysphagia severity will be assessed.

Any (serious) adverse events ([S]AEs) will be monitored, evaluated, and documented. The relation of (S) AEs to the test product will also be evaluated by a medical doctor. Specific safety outcomes of the current study are: respiratory tract infection, urinary tract infection, dehydration, pressure ulcer, fall incident, all-cause hospitalization, and choking. An overview of study outcomes, corresponding instruments and assessment details can be found in Table 5.

**Table 5:** Overview of study outcomes, instruments, and assessment details

	***Outcome***	***Instruments***	***Score***	***Assessment details***
***Primary***	Body Weight	Calibrated lift with integrated scale, mobile chair with integrated scale or wheelchair platform with integrated scale	Body weight weighed to the nearest gram	The resident is in fasting state and preferably dressed in light clothing and without shoes
***Secondary***	Nutritional intake	Standardized three-day food diary and a kitchen scale	Food products weighed to the nearest gram	Food products, leftovers and napkins with spit out food pieces will be weighed by a member of the trained study team
	HRQoL and OD-specific QoL	Standardized questionnaire EQ-5D-5L [33] and VAS: EQ VAS, DSS, DHRQoL [34, 35] and DAS	EQ-5D descriptive system [no problems/slight problems/moderate problems/severe problems/extreme problems] EQ VAS [0–100 score], DSS [0–10 score], DHRQoL [0–10 score], DAS [0–10 score]	Self-assessment or proxy-assessment
	Compliance to test product	Standardized compliance log	Amount of product leftover [no leftover/quarter/half/three quarters/complete leftover]	Filled out by nursing staff
***Exploratory***	ADL	Validated Barthel Index (BI) [36]	0 [dependent] - 20 [independent]	Conducted by a member of the trained study team
	Activity	Activity monitor activPAL3™ (PAL Technologies Ltd., Glasgow, UK) [37, 38] with activity log	Steps [count], upright/sitting/lying position [hours], stepping/standing position [hours]	Applied and completed by a member of the trained study team
	Anthropometrics	Measuring tape	Upper-arm and calf-circumference [cm]	Conducted by a member of the trained study team
	Blood nutritional- and hydration parameters	Laboratory analysis	Osmolality [mOsmol/kg], sodium [mmol/L], vitamin D [nmol/L], vitamin B12 [pmol/L], folate [nmol/L], magnesium [mmol/L], albumin (g/L) and calcium [mmol/L]	Clinical chemical and hematological laboratory of the Zuyderland Hospital (Heerlen-Sittard, the Netherlands)
	Vital signs	Electronic blood pressure monitor	Heart rate [bpm] and systolic and diastolic blood pressure [mmHg]	Conducted by a member of the trained study team
	Oxygen saturation	Oximeter	Oxygen saturation level [%]	Conducted by a member of the trained study team
	Dysphagia severity: swallowing assessment	3-oz WST [26], swallow observation, water thickness test, Modified version of the MISA [27]	Swallow of 3 oz water [positive/negative], descriptive report summary of swallow observation, safest thickness level of liquids for swallowing, completed modified MISA	Conducted by the trained SLT More details in Table 3
	Palatability of the test product	Standardized questionnaire	Taste [1 (poor taste) - 10 (very tasty)], viscosity [too thick/good/too thin], sweetness [too sweet/good/not sweet enough] and swallowing ability [1 (very difficult) - 10 (very easy)]	Self-assessment
***Safety***	(S)AEs	(S) AE forms	MedDRA Medical Coding	Coding will be done by the data manager

*Abbreviations*: EuroQol 5-dimensions 5-level (EQ-5D-5L), Visual Analog Scale (VAS), EUROQoL-VAS (EQ-VAS), Dysphagia Severity Scale (DSS), Dysphagia HRQoL (DHRQoL), Dysphagia-related Anxiety Scale (DAS), 3 oz Water Swallow Test (3-oz WST), McGill Ingestive Skills Assessment (MISA), (Serious) Adverse Event ((S)AE)

### Monitoring and data management

All procedures and persons involved in the collection, handling, and storage of data and documents will be registered in a delegation log. Monitoring will be done by the Clinical Trial Centre Maastricht (CTCM). The data will be handled according to the EU General Data Protection Regulation (GDPR) and the Dutch Act on implementation of the GDPR. Encoded data will be stored in an electronic case report form (eCRF) hosted by an external vendor (Viedoc by PCG Solutions, Uppsala, Sweden).

### Power calculation

Previous clinical trials on ONS in nursing home residents with malnutrition risk have shown a significantly increased body weight in residents that received ONS over a period of three to 6 months compared to residents receiving dietary recommendations or standard OD and nutritional care during this period [21–24]. In a large meta-analysis, Milne et al. (2009) [21] reported a significant weighted mean difference of 2.65% in body weight between patients with mixed geriatric conditions at risk of malnutrition using energy supplementation versus patients without energy supplementation. This equals an effect difference of ~ 1.4 kg for a person weighing 55 kg. This is in line with more recent studies reporting body weight change from baseline of 1.2 kg (SD 2.4) [24], 1.22 kg (SEM 0.45) [23], and 1.4 kg (SD 2.4) [39] after supplementation with ONS (500–600 kcal/day) for 8–12 weeks in nursing home residents with malnutrition risk. Therefore, an increase of 1.4 kg (SD 2.4) in body weight is assumed to be a reasonable expected mean outcome for the current test group. The mean body weight change for the control group is conservatively estimated to remain unchanged despite standard OD and nutritional care. Stange et al. (2013) [24] documented fourteen drop-outs from 87 residents (16%) in the test group. For the current study, we estimate a drop out percentage after randomization of 20%. Based on this a priori estimate with an alpha of 0.05 and 0.90 power, of the required sample size per group (control/ test) was made and totals 78.

### Data analysis

SAS/STAT® Software (by SAS Institute, North Carolina, USA) will be used for analysis of the encrypted data. Descriptive statistics of numerical data will be presented using mean and standard deviation (SD) or median with interquartile range (IQR), and they will be checked visually for normality using histograms and Q-Q plots. Categorical data will be presented in terms of frequencies or percentages. Numerical data of compliance to the test product will also be dichotomized into compliant and not-compliant. Generally, all analyses will be performed according to the intention-to-treat (ITT) principle. Only residents compliant to the intervention will be included in the per protocol analysis, which will be used as a sensitivity analysis for the ITT analyses. Linear mixed models will be performed to check for differences in primary and secondary outcome parameters between the control and the test group at different time-points with correction for baseline differences. A linear mixed model analysis will be used, as this method uses all available data, deals with correlation between repeated measures and assumes missingness to be at random.

### State of the study

The study was initiated on the 22nd of August, 2019. The study will proceed with inclusion after Covid-19 and reopening of the centers.

## Discussion

Due to the impairment of oral intake and swallowing, residents with OD are more prone to becoming malnourished [13]. Nutrient density of solid food decreases as water is added to blend the food product, [40] which in turn could increase the feeling of satiety without sufficient caloric intake. In addition, nutrition intake is further hampered because texture modification of solid food products and thickening of liquids with thickening powder may affect appetite and patient compliance [18, 19]. Hence, providing best care for this specific group of patients requires an interdisciplinary approach. The search for available and appropriate nutritional care for dysphagic residents is still ongoing, and the need for tailored and practical solutions is becoming even more pressing due to the aging of the population [13].

The results of the DYNAMO study will help to enhance best care for nursing home residents with OD and malnutrition (risk). Best care encompasses the promotion of the safety and efficacy of swallowing and the optimization of the nutritional status. This study is the first study on the effect of pre-thickened ONS on nutritional status in a vulnerable group of nursing home residents with OD and malnutrition (risk).

We hypothesize that the test group will show an improved change of body weight compared to the control group after 12 weeks of intervention, based on results of previous studies with ONS supplementation in nursing home residents [21–24]. The effect is expected within a period of 12 weeks, meaning adjacent policy in care can be determined within this period. An extended study period will lead to unnecessary burden to the participants and a delay of the implementation of the intervention in standard care.

In addition, the previous studies were conducted in nursing home residents who did not specifically have OD and who were supplemented with regular ONS, i.e. not specifically designed and likely not suitable for residents with OD. Providing a pre-thickened ready-to-use amylase-resistant ONS with a consistency that suits the severity levels of OD could improve swallowing efficacy and safety and oral intake compliance in nursing home residents with OD and malnutrition (risk). This in turn could have positive effects on the nutritional status. The current study therefore also includes QoL, ADL, dietary intake, intake compliance to the test product, and other parameters related to nutritional status. Associations between these parameters will be assessed since improving nutritional status with ONS may further lead to improved QoL and ADL [41].

Practical implementation of the DYNAMO study was closely aligned with nursing home staff and experts in the field of OD and malnutrition. Assessment tools and measurements used in the current study are based on standard OD and nutritional care where possible. For example, SNAQ-RC was used for screening of malnutrition (risk) as this is the standard tool for residential care in the Netherlands. Dutch national OD guidelines recommend bedside screening assessments like a WST and a clinical observation of oral intake [16, 42]. Although the psychometric limitations of these methods are known, instrumental swallowing assessments with a higher sensitivity and specificity, i.e. videofluoroscopy of swallowing (VFS) or fiberoptic/flexible endoscopic evaluation of swallowing (FEES) [13], have practical limitations and are therefore not part of the standard nursing home care. In the current study, OD screening will be carried out using a combination of existing practice (WST/water thickness test and swallowing observation) and a simplified version of a validated evaluation of functional ingestive skills of older persons (MISA) in order to obtain a patient-tailored recommendation on the safest thickness level for liquids.

Practical implementations and ethical considerations made it impossible to blind participants and assessors in this study. To the best of our knowledge, we have made every effort to limit the effects of this limitation. Participants are randomly allocated to a treatment arm and intervention procedures (apart from the intervention) are standardized for both treatment groups. The primary measure of the study is an objective and valid measure, all measurements are in line with standard care practice and possible effects will be considered in the analysis of the study outcomes.

This pragmatically controlled study closely reflects standard OD and nutritional care, and study outcomes therefore have real-life applicability. Execution of standard care in the study design also entails a number of limiting factors. Most importantly, standard care may differ slightly between nursing homes, and proper study execution is highly dependent on the engagement of all involved healthcare professionals. Both somatic and psychogeriatric nursing home residents will be included, since OD and malnutrition (risk) are common in both populations. This will result in a highly heterogeneous population, increasing outcome variability. In addition, including incapacitated residents limits some assessments or limits some assessments to be completed by a caretaker. A priori estimation of the inclusion rate is difficult, specifically, the informed consent rate, i.e. the willingness of residents or their legal representatives to participate. Hence, the inclusion rate will be closely monitored.

Dutch nursing homes often comprise of long-term somatic and psychogeriatric wards and short-term rehabilitation wards. Residents at rehabilitation wards follow a rehabilitation trajectory (e.g. after stroke). They are temporarily in need of high intensity care and leave the nursing home after a short period of time. Future studies could also target a group of rehabilitation residents with OD and concurrent malnutrition (risk). In addition, the body of literature on non-institutionalized older populations at risk for OD and malnutrition is poor.

## Data Availability

Study datasets are available from the corresponding author upon reasonable request. By signing informed consent, residents or their legal representatives give permission to the study team to share encoded data with the subsidizing party. Study results will be published by the investigating party in a scientific journal.

## Abbreviations

AE: Adverse Events
BI: Barthel Index
BDA: British Dietetic Association
BMI: Body Mass Index
CTCM: Clinical Trial Centre Maastricht
DAS: Dysphagia related Anxiety Scale
DHRQoL: Dysphagia related HRQoL
DSS: Dysphagia Severity Scale
ESPEN: European Society for Clinical Nutrition and Metabolism
EQ-5D-5L: EuroQol 5-dimensions 5-level
EQ-VAS: EuroQol Visual Analog Scale
FEES: Fiberoptic Endoscopic Evaluation of Swallowing
GDPR: General Data Protection Regulation
HRQoL: Health-Related Quality of Life
IDDSI: International Dysphagia Diet Standardization Initiative
IQR: Interquartile Range
ITT: Intention-to-treat
ICH-GCP: International Conference on Harmonization Good Clinical Practice
METC: Medical Ethics Committee
MISA: McGill Ingestive Skills Assessment
NTR: Dutch (Nederlands) Trial Register
OD: Oropharyngeal Dysphagia
ONS: Oral Nutritional Supplementation
NUTRIM: School of Nutrition and Translational Research in Metabolism
SPIRIT: Standard Protocol Items: Recommendations for Interventional Trial
SLT: Speech and Language Therapist
SAE: Serious Adverse Event
SNAQ-RC: Short Nutritional Assessment Questionnaire for Residential Care
SD: Standard Deviation
V-VST: Volume-Viscosity Swallow Test
VFS: Videofluoroscopy
VAS: Visual Analog Scale
WST: Water Swallow Test
WMO: Dutch Act for Medical Research Involving Human Subjects

## Footnotes

1 ‘DYNAMO’ is an acronym for this randomized controlled trial (RCT) comprising of Oropharyngeal Dysphagia, Malnutrition and Nursing and is Greek for life and vital force.
